# Distribution patterns and variation analysis of simple sequence repeats in different genomic regions of bovid genomes

**DOI:** 10.1038/s41598-018-32286-5

**Published:** 2018-09-26

**Authors:** Wen-Hua Qi, Xue-Mei Jiang, Chao-Chao Yan, Wan-Qing Zhang, Guo-Sheng Xiao, Bi-Song Yue, Cai-Quan Zhou

**Affiliations:** 1 College of Biology and Food Engineering, Chongqing Three Gorges University, Chongqing, 404100 P. R. China; 2 College of Environmental and Chemistry Engineering, Chongqing Three Gorges University, Chongqing, 404100 P. R. China; 3 Key Laboratory of Bio-resources and Eco-environment (Ministry of Education), College of Life Sciences, Sichuan University, Chengdu, 610064 P. R. China; 4 College of Life Sciences, Sichuan Agricultural University, Ya′an, Sichuan Province 625014 P. R. China; 5 Key Laboratory of Southwest China Wildlife Resources Conservation (Ministry of Education), China West Normal University, Nanchong, 637009 P. R. China

## Abstract

As the first examination of distribution, guanine-cytosine (GC) pattern, and variation analysis of microsatellites (SSRs) in different genomic regions of six bovid species, SSRs displayed nonrandomly distribution in different regions. SSR abundances are much higher in the introns, transposable elements (TEs), and intergenic regions compared to the 3′-untranslated regions (3′UTRs), 5′UTRs and coding regions. Trinucleotide perfect SSRs (P-SSRs) were the most frequent in the coding regions, whereas, mononucleotide P-SSRs were the most in the introns, 3′UTRs, TEs, and intergenic regions. Trifold P-SSRs had more GC-contents in the 5′UTRs and coding regions than that in the introns, 3′UTRs, TEs, and intergenic regions, whereas mononucleotide P-SSRs had the least GC-contents in all genomic regions. The repeat copy numbers (RCN) of the same mono- to hexanucleotide P-SSRs showed significantly different distributions in different regions (*P* < 0.01). Except for the coding regions, mononucleotide P-SSRs had the most RCNs, followed by the pattern: di- > tri- > tetra- > penta- > hexanucleotide P-SSRs in the same regions. The analysis of coefficient of variability (CV) of SSRs showed that the CV variations of RCN of the same mono- to hexanucleotide SSRs were relative higher in the intronic and intergenic regions, followed by the CV variation of RCN in the TEs, and the relative lower was in the 5′UTRs, 3′UTRs, and coding regions. Wide SSR analysis of different genomic regions has helped to reveal biological significances of their distributions.

## Introduction

Microsatellites (or Simple sequence repeats, SSRs) are composed of tandem repeats of 1–6 oligonucleotides. It has been reported that SSRs play an important role in chromatin fractions, gene expression and regulation, as well as transcription and protein function^1,2^. They are hypermutable loci due to strand slippage and unequal recombination lead to indels of repeat units^3^, which affect local structure of the DNA or protein sequences^2^. Variation of intronic SSRs can affect gene transcription and mRNA splicing^4^; Trinucleotide SSRs located in the UTRs (untranslated regions) or introns can also induce gene silencing^4^. Distribution of SSRs in the coding regions, 5′UTRs, introns, and 3′UTRs of genes are widely belived to affect transcription and translation as well as gene function^4^. The increase and decrease of SSR motifs in the 5′UTRs are known to regulate multiple characteristics^5–7^. Tri- and hexanucleotide SSRs in genes encode into amino acid, which may play particular roles in protein structure^8,9^. SSRs in both coding and regulatory regions can alter the structure of proteins or DNA when they expand beyond a certain length^10^.

*In silico* mining and analysis of SSRs could help to disclose different aspects of the distribution and dynamics of SSRs in eukaryotic genomes^11^. There are two SSR search methods: using a suitable search tool (MISA^12^, SciRoKo^13^, msatcommander^14^, GMATA^15^, Krait^16^) and accessing a relevant SSR database (MMDBJ, SSRD, TRBase, InSatdb, and TRDB)^11^. The mining and analysis of SSRs not only helps in addressing biological questions, but also facilitates better utilizing of SSRs for multiple utilizations. The genome sequence data from six bovid species: *Bos taurus*, *Bos mutus*, *Bubalus bubalis*, *Ovis aries*, *Capra hircus*, and *Pantholops hodgsonii*, were used in this study. We detected and characterized SSRs and their motifs, and surveyed their distributions and variations in intragenic (i.e., 5′UTRs, coding regions, introns, and 3′UTRs) and intergenic regions. Furthermore, we addressed the questions of whether the abundance of different SSR types and motifs are similar or not in different genomic regions and how GC-content of SSR differ in 5′UTRs, coding regions, introns, 3′UTRs, transposable elements (TEs, or transposon), and intergenic regions. This research may facilitate our insight into SSR distribution of different genomic regions in the whole genome and GC-content difference of mono- to hexanucleotide SSRs. Repeat copy number (RCN) can provide some markers for studying processes of mutation and selection. Intragenic- and intergenic-wide analysis of SSR sequences of different bovid species has also improved our understanding of biological significances of SSR distributions.

## Results

### Distribution of SSRs in different genomic regions of bovid genomes

In the 5′UTRs, coding regions, introns, 3′UTRs, TEs, and intergenic regions of these bovids, P-SSRs was the most frequent type, and the least was in the complex SSRs (CX-SSRs, Fig. S1); the intronic and intergenic regions had the most abundant P-SSRs, followed by the pattern: 3′UTRs > 5′UTRs > TEs > coding regions (Fig. S1). The relative abundance of the same SSR types showed greatly similar in the same regions of bovid species.

**Figure 1:**
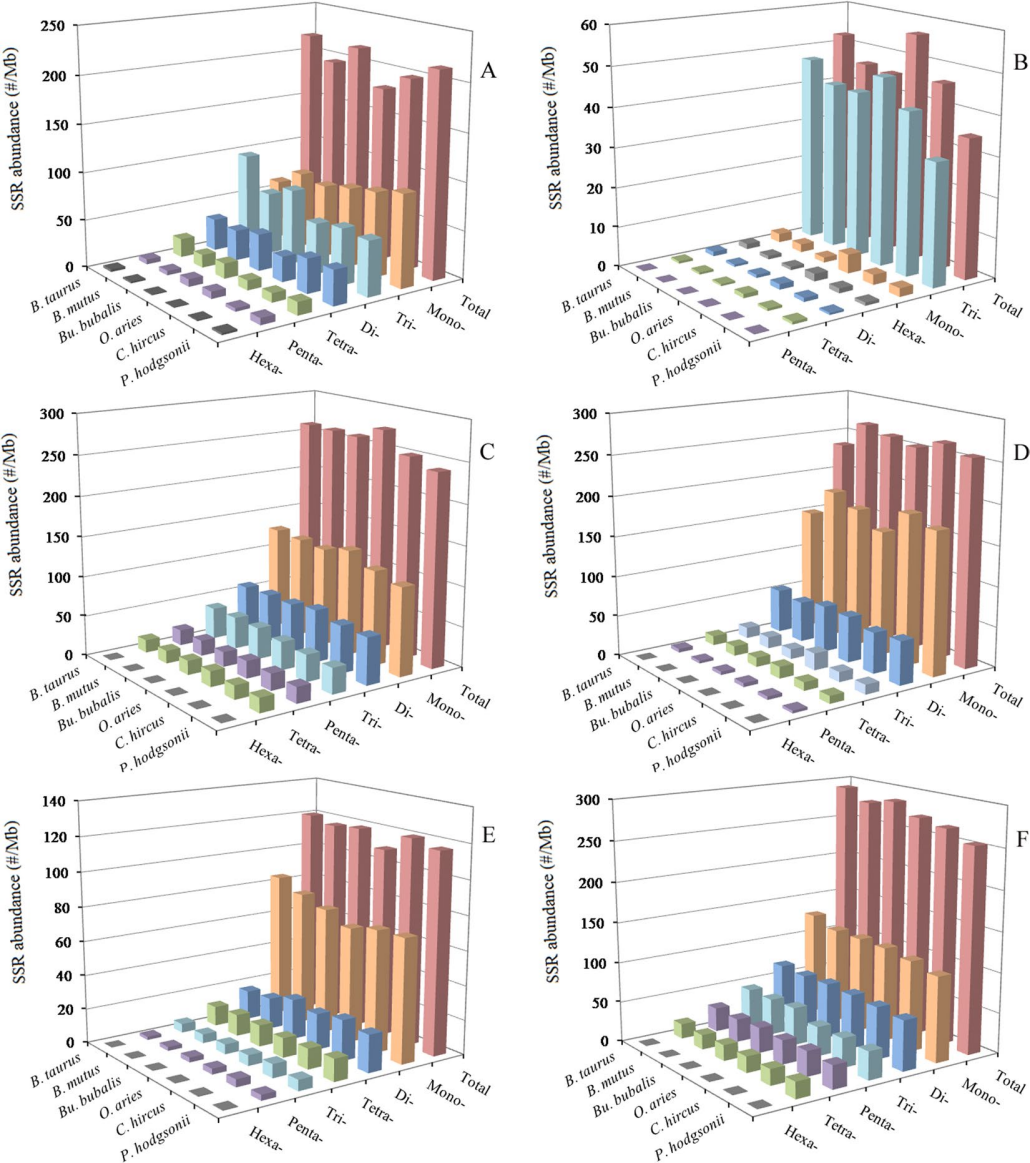
Relative abundance of mono- to hexanucleotide P-SSRs in different intragenic and intergenic regions of six bovids. ABCDEF represent 5′UTRs, coding regions, introns, 3′UTRs, TEs, and intergenic regions, respectively.

In the 5′UTRs, tri- and mononucleotide P-SSRs were the most frequent type, followed by the pattern: di- > tetra- > penta- > hexanucleotide P-SSRs in the six bovid species (Fig. 1A and Table S1). In the coding regions, trinucleotide P-SSRs was the most frequent type, followed by the pattern: mono- > hexa- > di- > tetra- > pentanucleotide P-SSRs in these bovid species (Fig. 1B and Table S2). Pentanucleotide P-SSRs were relatively less frequent in the coding regions of these bovid species. In the 3′UTRs, mononucleotide P-SSRs was the most frequent type, followed by the pattern: di- > tri- > tetra- > penta- > hexanucleotide P-SSRs, the least was in the hexanucleotide P-SSRs in these species (Fig. 1D and Table S4). In the TEs, mononucleotide P-SSRs was the most frequent type, followed by the pattern: di- > tetra- > tri- > penta- > hexanucleotide P-SSRs in the bovid genomes (Fig. 1E and Table S5). In the TEs, mononucleotide P-SSRs was more than three times as frequent as di- and tetranucleotide P-SSRs, and interestingly, the latter are much more frequent than trinucleotide P-SSRs. In the intronic and intergenic regions, mononucleotide P-SSRs was the most frequent type, followed by the pattern: di- > tri- > penta- > tetra- > hexanucleotide P-SSRs, the least was in the hexanucleotide P-SSRs in these bovid species (Fig. 1C, F, and Tables S3, S6). In the introns, mononucleotide P-SSRs were more than twofold as frequent as dinucleotide P-SSRs. Interestingly, in the intronic and intergenic regions pentanucleotide P-SSRs are much more frequent than tetranucleotide P-SSRs, and hexanucleotide P-SSRs were relatively less abundant.

**Figure 2:**
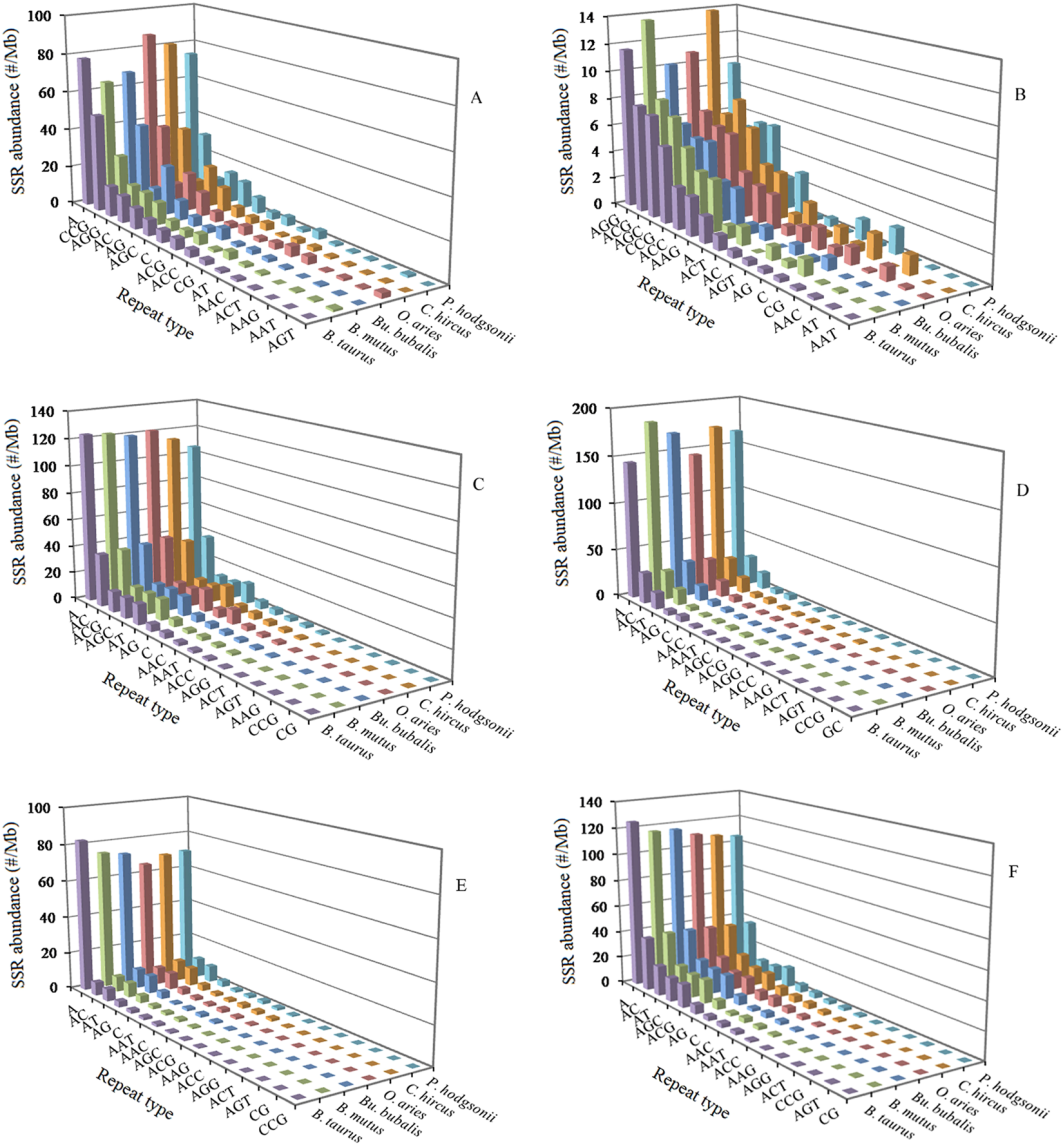
Distribution of different motfis of mono- to trinucleotide P-SSRs in different genomic regions of six bovid genomes. ABCDEF represent 5′UTRs, coding regions, introns, 3′UTRs, TEs, and intergenic regions, respectively.

A comparison among these regions shows that relative abundance of the same mono- to hexanucleotide P-SSRs showed great similarity in the same genomic regions of these bovid species. Remarkably, the total SSR abundance among all regions for these species is the most for the intergenic regions (Fig. 2). There are more than five times the difference between the total SSR abundance of the coding regions and intergenic regions. SSR distribution seems to be the similarity between intronic and intergenic regions of these bovid genomes. These results here indicated that SSRs are more frequent in non-coding regions than coding regions in these bovid species.

### Diversity of P-SSRs motifs in different genomic regions of bovid genomes

The abundance of different repeat motifs varied obviously with genomic regions in the six bovid species. In the 5′UTRs, the (A)_n_ was the most frequent motif, followed by the motif (CCG)_n_, thirdly the (AGG)_n_, (AC)_n_, and (AG)_n_, fourthly the (AGC)_n_ and (ACG)_n_ (Fig. 2A). In the coding regions, the (AGG)_n_ was the most frequent unit, followed by the motif (ACG)_n_, (AGC)_n_, and (CCG)_n_, thirdly the (ACC)_n_, (AAG)_n_, (A)_n_, and (ACT)_n_ (Fig. 2B). In the introns, the (A)_n_ was the most frequent unit, followed by the motif (AC)_n_, thirdly the (ACG)_n_, (AGC)_n_, and (AT)_n_, fourthly the (AG)_n_, (C)_n_, (AAC)_n_, (AAAT)_n_, and (AAAC)_n_, the (CG)_n_ and (CCG)_n_ were relatively infrequent in the intronic regions (Fig. 2C). In the 3′UTRs, the (A)_n_ was the most frequent motif, followed by the motif (AC)_n_, thirdly the (AT)_n_, fourthly the (AG)_n_ and (C)_n_ (Fig. 2D). In the TEs, the (A)_n_ was the most frequent motif, followed by the motif (AC)_n_ and (AT)_n_, thirdly the (AG)_n_ and (AAAT)_n_, fourthly the (C)_n_, (AAT)_n_, (AAC)_n_, (AGC)_n_, and (AAAC)_n_ (Fig. 2E). In the intergenic regions, the (A)_n_ was the most frequent motif, followed by the motif (AC)_n_, thirdly the (AT)_n_, (AGC)_n_, and (ACG)_n_, fourthly the (AG)_n_, (C)_n_, (AAAT)_n_, (AAC)_n_, and (AAAC)_n_ (Fig. 2F). Therefore, the motifs of SSRs are not randomly distributed in the 5′UTRs, coding regions, introns, 3′UTRs, TEs, and intergenic regions. There is a noticeable excess of (CCG)_n_ repeat units in the 5′UTRs and coding regions compared to the introns, 3′UTRs, TEs, and intergenic regions. The (AGG)_n_ repeat unit is obvious relatively abundant in the 5′UTRs and coding regions compared to other four regions. The (ACG)_n_ and (AGC)_n_ repeat units are relatively less abundant in the TEs compared to other five regions. The (A)_n_ motif was significantly more frequent than the (C)_n_ unit in the 5′UTRs, introns, 3′UTRs, TEs, and intergenic regions. The (AAT)_n_ and (AAC)_n_ units are relatively frequent in the TEs, where their abundance exceeds that of other trinucleotide motifs, and the (CG)_n_ and (CCG)_n_ motifs are relatively infrequent in the introns, TEs, 3′UTRs, and intergenic regions.

### The GC-content of P-SSRs in different genomic regions of bovid genomes

**Figure 3:**
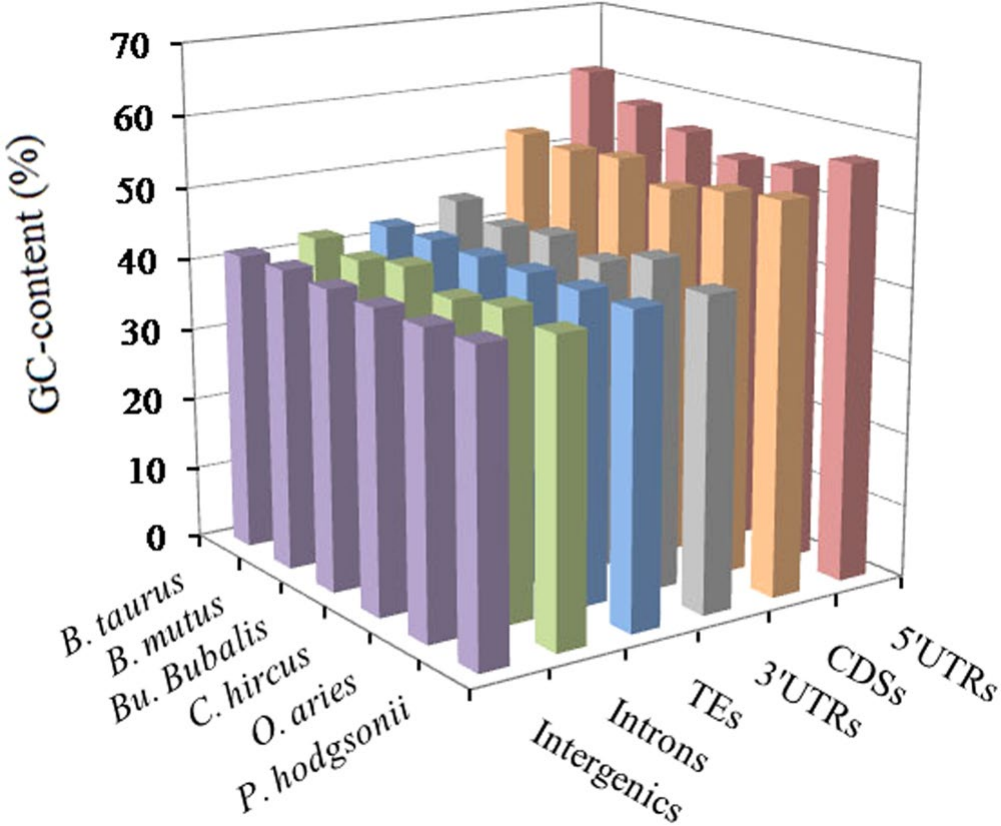
GC-contents of different intragenic and intergenic regions in six bovid species.

The GC-content varied greatly among different genomic regions, but, in the same regions, the distribution of the GC-content is greatly similar. From the results (Fig. 3), we can know that 5′UTRs had the most GC-content (ranging 53.75–61.31%), followed by the coding regions (51.09–53.60%), next the 3′UTRs (42.61–45.18%) and TEs (42.53–42.83%), the least was the intronic (40.87–42.91%) and intergenic regions (41.39–41.84%). The distribution patterns of AT-contents (adenine-thymine content) showed greatly similar in the same genomic regions of these bovids (Table S7). From this we can know, high GC-content was distributed in exon-rich regions more frequently than other regions.

**Figure 4:**
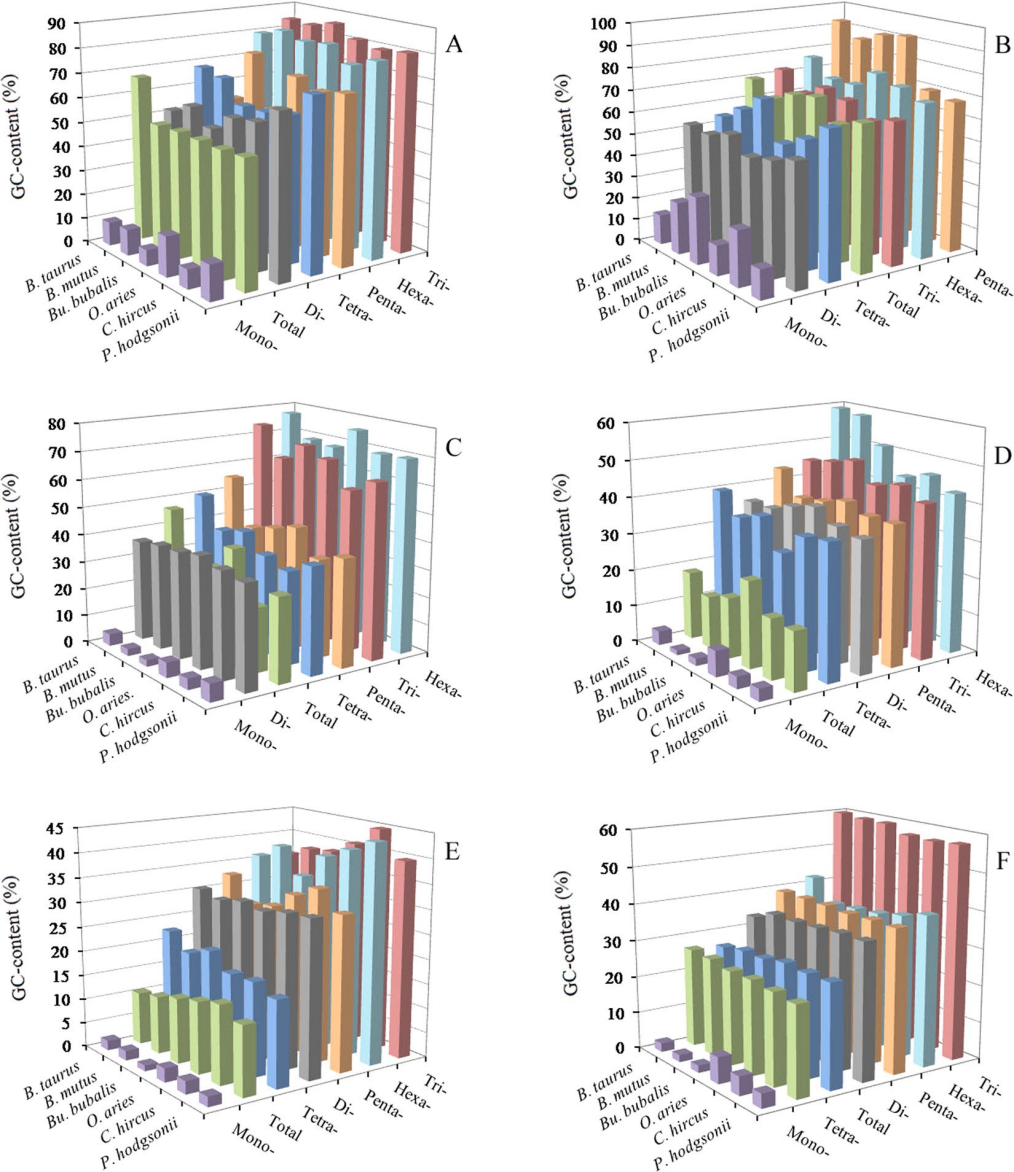
GC-contents of mono- to hexanucleotide P-SSRs in different intragenic and intergenic regions of six bovid species. ABCDEF represent 5′UTRs, coding regions, introns, 3′UTRs, TEs, and intergenic regions, respectively.

The AT- and GC-content of P-SSRs were calculated in the 5′UTRs, coding regions, introns, 3′UTRs, TEs, and intergenic regions of six bovid species, which the results were shown in Fig. 4 and Tables S8–13. In the six genomic regions, mononucleotide P-SSRs had the least GC-contents and were significantly less than their total GC-contents in these bovid genomes. In the 5′UTRs, except for the mononucleotide P-SSRs, the GC-content of the remaining nucleotide motifs are more than their AT-content (Fig. 4A and Table S8). Trinucleotide P-SSRs had the most GC-content (79.49–86.15%), followed by the pattern: hexa- > penta- (and tetra-) > di- > mononucleotide P-SSRs in the 5′UTRs of these bovid species (Fig. 4A). In contrast, the GC-content in the tri-, tetra- and hexanucleotide P-SSRs were more than their total GC-content in the 5′UTRs of these bovids (Fig. 4A). In the coding regions, the most GC-contents were in penta- and hexanucleotide P-SSRs, ranging from 68.00% (*P. hodgsonii*) to 92.80% (*B. taurus*), which were more than their AT-contents, and the GC-contents of mono-, di-, and tetranucleotide repeat types were significantly lower than their total GC-contents (61.67–70.58%) in these bovids, especially in mononucleotide P-SSRs (Fig. 4B and Table S9). In the 3′UTRs, except for the hexanucleotide P-SSRs, the GC-contents of the remaining nucleotide repeat units were less than their AT-contents, and mononucleotide P-SSRs had the least GC-contents (Fig. 4D and Table S11). In the intronic and intergenic regions, the most GC-contents were all in trinucleotide P-SSRs, followed by the pattern: penta- (and hexa-) > di- > tetra- > mononucleotide P-SSRs, and di-, penta-, and hexanucleotide P-SSRs are of similar GC-contents in the bovids (Fig. 4C, F and Tables S10, S13). In the TEs, we can know that the GC-contents of mono- to hexanucleotide P-SSRs are less than their AT-contents, and the most GC-contents were all in tri- and hexanucleotide P-SSRs, followed by the pattern: di- (and penta-) > tetra- > mononucleotide P-SSRs, di- and pentanucleotide P-SSRs are of similar GC-contents in these bovids (Fig. 4E and Table S12). In contrast, the GC-contents of di- to hexanucleotide P-SSRs were more than their total GC-contents in the 3′UTRs and TEs, and the GC-contents of di-, tri-, penta-, and hexanucleotide P-SSRs were also more than their total GC-contents in the intronic and intergenic regions. In the 3′UTRs, introns, TEs, and intergenoic regions, their total AT-contents ranged from 71.20% to 89.29%, were obviously higher than their total GC-contents; whereas, in the coding regions, their total GC-contents ranged from 61.67% to 70.58%, were obviously higher than their total AT-contents in the bovids. Therefore, the GC-content of P-SSRs is probably high in coding-rich regions, whereas, the AT-content of P-SSRs is probably quite high in non-coding regions of these bovids.

### The analysis of coefficient of variability (CV) of SSRs

**Figure 5:**
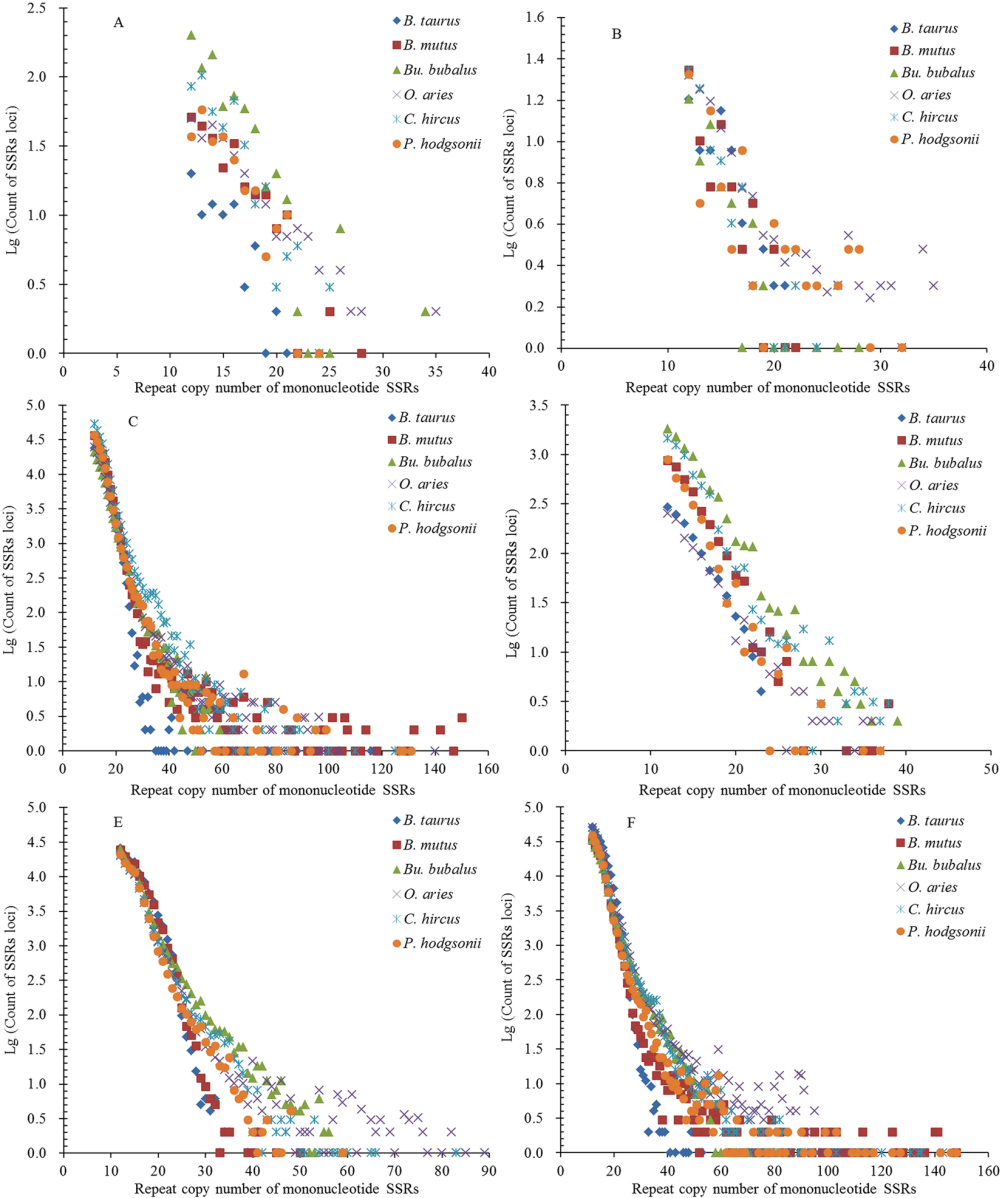
Comparative analysis of repeat copy number (RCN) of mononucleotide P-SSRs in different genomic regions of six bovid genomes. ABCDEF represent 5′UTRs, coding regions, introns, 3′UTRs, TEs, and intergenic regions, respectively.

**Figure 6:**
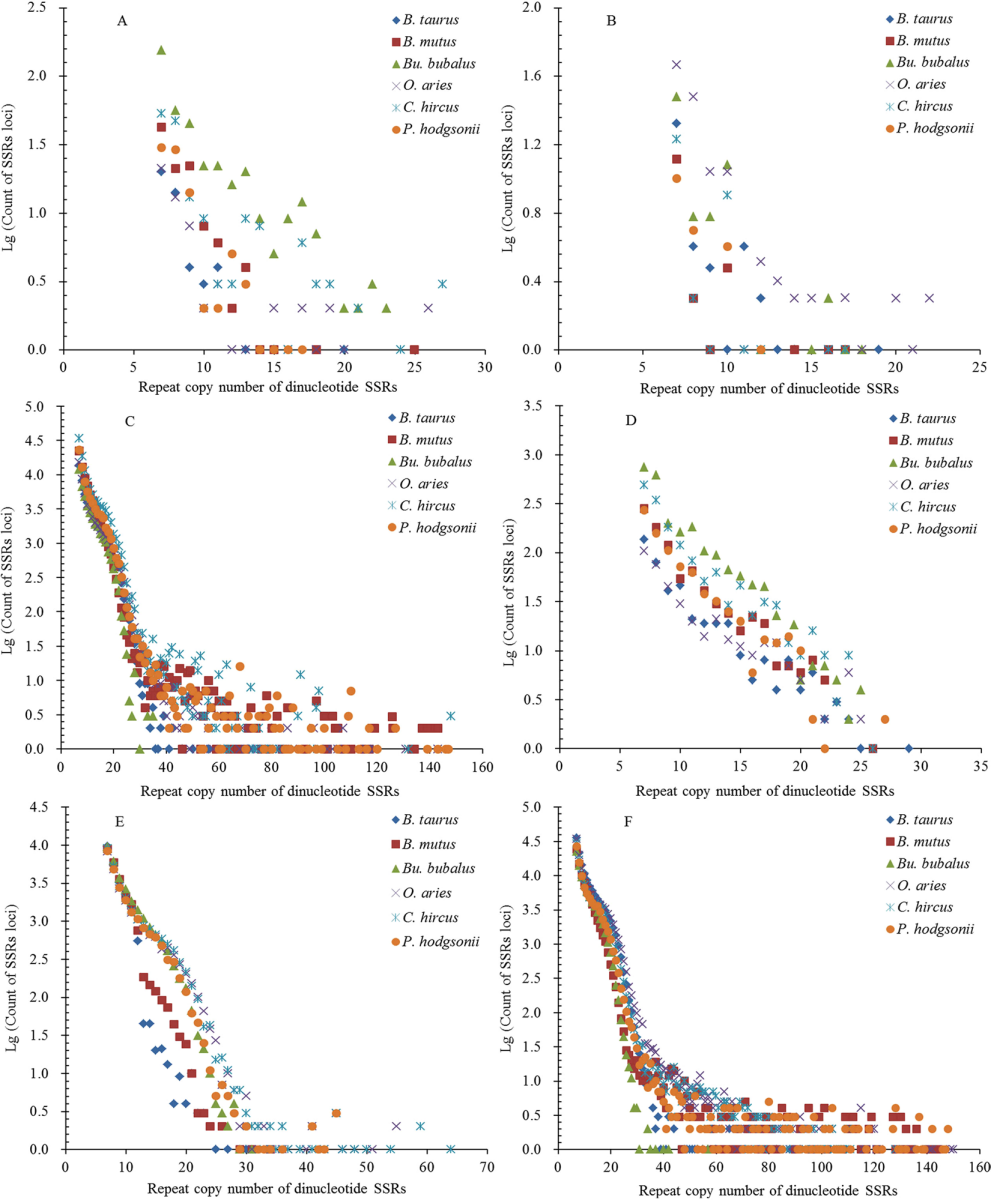
Comparative analysis of RCN of dinucleotide P-SSRs in different genomic regions of six bovid genomes. ABCDEF represent 5′UTRs, coding regions, introns, 3′UTRs, TEs, and intergenic regions, respectively.

**Figure 7:**
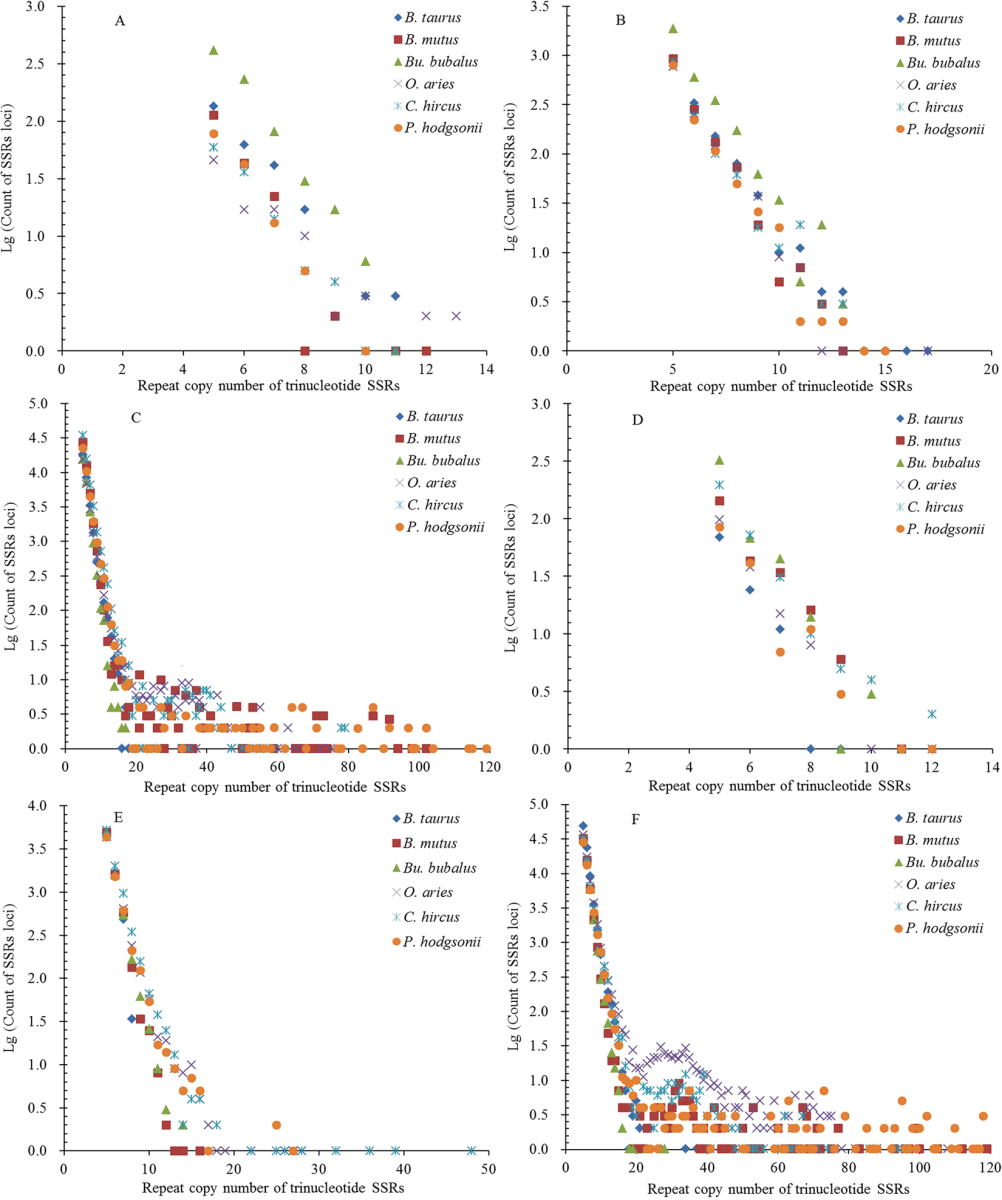
Comparative analysis of RCN of trinucleotide P-SSRs in different genomic regions of six bovid genomes. ABCDEF represent 5′UTRs, coding regions, introns, 3′UTRs, TEs, and intergenic regions, respectively.

The repeat copy numbers (RCN) of the same mono- to hexanucleotide SSRs had significantly different distributions in the different regions of these bovid genomes. The RCN of mono- and dinucleotide SSRs exhitbited great similar distributions and had the most counts of SSR loci in the intronic and intergenic regions, which were mainly distributed from 12 to 65 times and from 7 to 60 times, respectively (Fig. 5C, F and Fig. 6C, F). The RCN of mono- and dinucleotide SSRs were distributed from 10 to 60 times in the intronic and intergenic regions, which were clustered together and overlapped each other (Fig. 5C, F and Fig. 6C, F). The RCN of mono- and dinucleotide SSRs had the second most counts of SSR loci in the TEs, which were mainly distributed from 12 to 50 times and from 7 to 30 times, respectively. The RCN of mononucleotide SSRs were distributed from 12 to 40 times in the TEs, which were clustered together and overlapped each other (Fig. 5C, F and Fig. 6C, F). In the 3′UTR regions, the RCN of mono- and dinucleotide SSRs displayed great similar among different bovid species, which were mainly distributed from 12 to 40 times and from 7 to 30 times, respectively (Fig. 5D and Fig. 6D). The RCN of mono- and dinucleotide SSRs had the fewest counts of SSR loci in the 5′UTRs and coding regions, which were mainly distributed from 12 to 30 times and from 7 to 20 times, respectively (Fig. 5A, B and Fig. 6A, B). The RCN of trinucleotide SSRs also showed great similar distributions and had the most counts of SSR loci in the intronic and intergenic regions, which were all mainly distributed from 5 to 40 times. The RCN of trinucleotide SSRs were distributed from 5 to 20 times in the intronic and intergenic regions, which were clustered together and overlapped each other (Fig. 7C, F). The RCN of trinucleotide SSRs had second most counts of SSR loci in the TEs, which were mainly distributed from 5 to 20 times (Fig. 7E). The RCN of trinucleotide SSRs had the fewest counts of SSR loci in the 5′UTRs, coding regions, and 3′UTRs, which were mainly distributed from 5 to 12 times (Fig. 7A, B, D).

The RCN of tetra- and pentanucleotide SSRs had most counts of SSR loci in the intronic and intergenic regions, which were mainly distributed from 4 to 30 times (Fig. S2C, F and Fig. S3C, F). The RCN of tetra- and pentanucleotide SSRs also showed great similar distributions and had second most counts of SSR loci in the TEs, which were mainly distributed from 4 to 12 times (Fig. S2E and Fig. S3E). The RCN of tetra- and pentanucleotide SSRs had fewer counts of SSR loci in the 5′UTR and 3′UTR regions, which were all mainly distributed from 4 to 6 times (Fig. S2A, D and Fig. S3A, D). The RCN of tetra- and pentanucleotide SSRs had fewest counts of SSR loci in the coding regions, which were mainly distributed from 4 to 5 times (Fig. S2B and Fig. S3B). The RCN of hexanucleotide SSRs had most counts of SSR loci in the intronic and intergenic regions, which were mainly distributed from 4 to 15 times (Fig. S4C, F). The RCN of hexanucleotide SSRs had second most counts of SSR loci in the TEs, which were mainly distributed from 4 to 9 times (Fig. S4E). The RCN of hexanucleotide SSRs were usually less and had fewer counts of SSR loci in the 5′UTRs, 3′UTRs, and coding regions, which were mainly distributed from 4 to 6 times (Fig. S4B).

**Figure 8:**
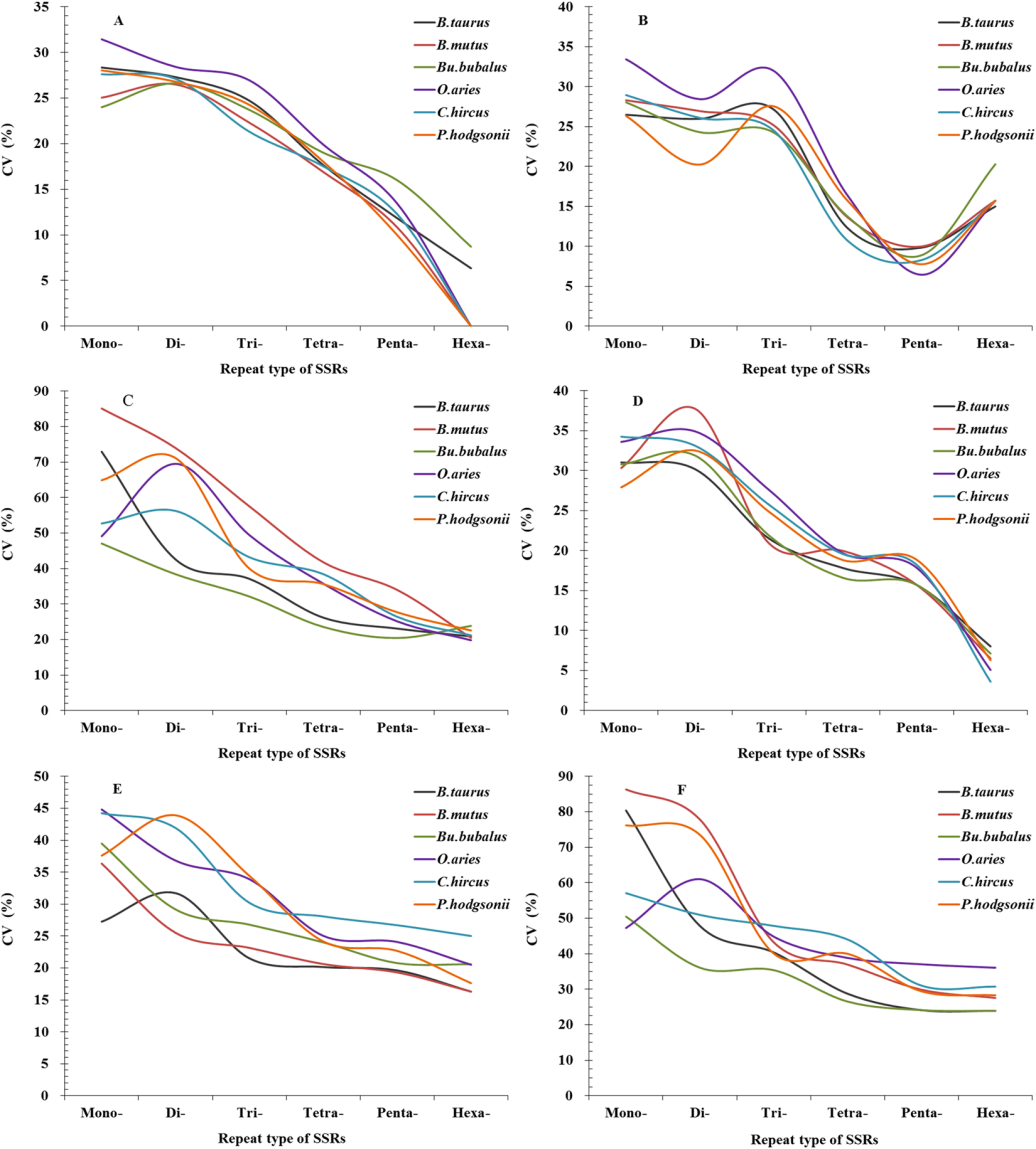
The CV analysis of RCN of SSRs in different genomic regions of six bovid genomes. ABCDEF represent 5′UTRs, coding regions, introns, 3′UTRs, TEs, and intergenic regions, respectively.

The analysis of coefficient of variability (CV) of SSRs showed that the RCN of mono- and dinucleotide SSRs had relative higher variation in the 5′UTRs, 3′UTRs, TEs, introns, and intergenic regions of the same bovid species, followed by the CV pattern of RCN: trinucleotide SSRs > tetranucleotide SSRs > pentanucleotide SSRs > hexanucleotide SSRs (Fig. 8). In the coding regions, the RCN of mono- to trinucleotide SSRs had relative higher variation, followed by the CV pattern of RCN: hexanucleotide SSRs > tetranucleotide SSRs > pentanucleotide SSRs (Fig. 8). The CV variations of the same mono- to hexanucleotide SSRs showed a great deal of similarity in the 5′UTRs, 3′UTRs, and coding regions of these bovid genomes, which also showed similar in the intronic and intergenic regions, whereas they are slightly different from the CV variations of the same SSRs in the TEs (Fig. 8). The CV variations of RCN of the same mono- to hexanucleotide SSRs were relative higher in the intronic and intergenic regions, followed by the CV variation of RCN in the TEs, and the relative lower was in the 5′UTRs, 3′UTRs, and coding regions (Fig. 8). It has been inferred that SSR mutational rates within genes are inconsistent with those for SSRs located in other genomic regions.

## Discussion

### Similarity and diversity of P-SSR motifs in different genomic regions

It was presumed that SSR motifs were not distributed randomly in the different genomic regions and motif types may play important roles in gene expression and regulation^17–20^. The presence of SSRs in different genomic regions shows bias to some specific nucleotide motifs. The motifs of mono- to hexanucleotide P-SSR types showed distinct distributional patterns in the intragenic and intergenic regions of bovid species. In *drosophila*, coding regions exhibit a very high bias to (AGC)_n_, and very rare for (TGC)_n_^21^. In the study, there is also a noticeable excess of (AGG)_n_ repeat units, and the second most frequent units are constituted by the (ACG)_n_, (AGC)_n_, and (CCG)_n_ in the coding regions compared to other genomic regions in the bovid species. The (CG)_n_ are relatively frequent in the 5′UTRs, whereas their abundance are very little in the coding regions, introns, 3′UTRs, TEs, and intergenic regions of the bovid species, this is consistent with the intragenic and intergenic regions of primates^22^. The (A)_n_ repeat units are the most abundant motifs in the 5′UTRs, introns, 3′UTRs, TEs, and intergenic regons of these bovid species, this is consistent with bovid geomes^23^. The second most frequent motifs are dinucleotide (AC)_n_ repeats in the introns, 3′UTRs, and intergenic regions of these bovid species, this is consistent with previous reports^22,23^. (ACG)_n_ and (AGC)_n_ motifs are comparatively frequent in intronic and intergenic regions of these bovid species, where their occurrence exceeds that of other trinucleotide repeat units. The (CCG)_n_ motifs are the most abundant repeat units in 5′UTRs, the second in the coding regions; whereas the (CCG)_n_ motifs are relatively infrequent in the introns, TEs, and intergenic regions, and also their abundance were less than that of other trinucleotide motifs in the bovid species. This is consistent with the different genomic regions of primates^22^. It has been demonstrated that the (CCG)_n_ motif was significantly presented in the upstream regions of the genes^24^. The distributional pattern of SSR motifs in different genomic regions may be correlated with the present frequency of certain amino acids.

### The variation of SSR abundance in different intragenic and intergenic regions

It has recently been reported that the distribution of SSRs is nonrandom in the genome, and their abundances vary widely in different genomic regions^22^. Recent evidences have demonstrated that SSRs in different genomic regions play different functional roles. Many SSRs exist in ORFs of higher eukaryotes^21,25–27^. Consistent with previous studies in primates and plants^22,27^, SSR abundance differs in 5′UTR and 3′ UTR regions of these bovid genomes. In the primates, trinucleotide SSRs show around double greater frequency in the 5′UTRs than that in the coding regions, whereas the latter had much more frequent trinucleotide P-SSRs than that in the intron, 3′UTRs, TEs^22^. Dominance of trinucleotide SSRs over other nucleotide units in coding regions may be caused by frameshift mutations to suppress non-trimeric SSRs in coding regions^28^. In *Arabidopsis thaliana*, low SSR abundances occurred in the centromeric region^29^. In *Drosophila melanogaster*, SSR distribution differs between X-chromosomes and autosomes^30^. Inconsistent with previous report^25,27^, the distributions of SSRs showed great similarity in the intronic and intergenic sequences of these bovid genomes. These reports suggest a significant heterogeneity of SSR distribution in different genomic regions of organism genomes.

It has been reported that changes of SSRs are involved in several human diseases^31–33^. Our results showed that the abundance of different SSR motifs varies with the genomic regions. SSRs have been shown to be more abundant in non-coding regions than that in coding regions^21,25,27,34^. In the different genomic regions of the same bovid species, the introns, 3′UTRs, and intergenic regions had the most abundant P-SSRs, followed by the pattern: 5′UTRs > TEs > coding regions. There seem to be no distinct differences in P-SSR abundance between intronic and intergenic regions, which is consistent with previous report^25^. P-SSR abundance is the least in the coding regions, suggesting that low SSR abundance may decrease the evolvability of proteins. This may be related to the fact that SSR births/deaths were strongly selected against in coding regions^35^.

This evidence has been proved that the mutations of coding regions could cause protein functional changes, loss of function, and protein truncation^4^. In different repeat type of these bovid species, trinucleotide P-SSRs were the most abundant type in the coding regions, whereas mononucleotide P-SSRs were the most frequent type in the 5′UTRs, introns, 3′UTRs, TEs, and intergenic regions; pentanucleotide P-SSRs were the least in the coding regions, whereas hexanucleotide P-SSRs were the least in the 5′UTRs, introns, 3′UTRs, TEs, and intergenic regions. In *Brassica rapa*, Trinucleotide SSRs were also the most frequent type in the coding regions^36^. In the exon regions, mononucleotide P-SSRs were the most abundant, followed by the pattern: tri- di- > tetra- > penta- > hexanucleotide SSRs in these bovid species. The abundances of hexanucleotide P-SSRs were less in the introns than that in the exons in these bovid species, which was inconsistent with previous reports^25^. It has been reported that coding regions are preferentially selected with trifold nucleotide SSR motifs^7,37–40^ and suppressed non-trimeric SSR repeat units, which can reduce potential translational frameshift mutations^28^. This evidence can contribute to explain why trifold nucleotide SSR repeat units are more frequent in coding regions than that in other genomic regions.

### The distributional pattern of GC-content in different genomic regions

Nucleotide composition influences SSR abundance, thus, the GC-content was examined in different genomic regions of six bovid species. The GC-contents of six bovid genomes showed to be remarkably consistent, but GC-contents varied greatly among different genomic regions. In this study, 5′UTRs had the most GC-content, followed by the coding regions (51.09–53.60%), thirdly the 3′UTRs and TEs, the least was the intronic and intergenic regions. Thus we can know that high GC-content was frequently distributed in exon-rich regions, and the distribution of GC-content was uneven in the bovid genomes. This evidence was consistent with the GC-content distributional pattern of different genomic regions in the primates^22^. Different classes of TEs tend to have bias for either GC-rich or GC-poor regions^41^. Ancestral Alu sequences have a high GC-content^42,43^. In the study, the repeat units of GC-richness were present in the 5′UTRs and coding regions, in which the GC-content were much higher than that in the remaining genomic regions (Fig. 4); whereas the motifs of AT-richness were present in the introns, 3′UTRs, TEs, and intergenic regions, in which the AT-content were much higher than that in the 5′UTRs and coding regions (Tables S8–13). It has recently been reported that top SSR motifs have a direct positive relationship with the GC- or AT-content in different genomic regions^44^. In contrast, the gradient of average GC-content decreases from the 5′UTRs to intronic regions by several percent to around 14.88% in these different genomic regions of the bovids. It has been reported that there is a gradient in the GC-content of Gramineae genes^45^. It has also been reported that SSR polymorphism was negatively correlated with the GC-content of the flanking regions of SSR locus^46^. Furthermore, the GC-content of different genomic regions in the genome could be used as a relative measure of mutation rate.

### Association of SSRs with other sequence elements and their mutability

SSRs associate characteristically with different intragenic and intergenic regions in the genome. SSR abundance is considerably high in 5′UTRs of plant genes^47,48^ and are relatively low in exonic sequences^47^. SSRs are richly distributed in the 5′UTRs, introns, 3′UTRs, and intergenic regions of primates, and are relatively few in the coding regions^22^. SSR distribution in introns is similar to that of the whole genome^22,47^. Genomic regions of SSR collection have been recognized in *Arabidopsis thaliana*^49^, *Drosophila melanogaster*^50^, and primates^22^. In 42 prokaryotic genomes, SSR distributions in coding regions were biased toward coding termini^51^. SSRs are also frequently found in the proximity of TEs^52–54^. It has been confirmed that SSRs are often associated with retrotransposons^55^, Alu elements, SINEs (short interspersed elements)^56^, MITES (miniature inverted transposable elements)^47,55^. (GAA)_n_ were associated with Alu repeats^56^. Abundant trinucleotide SSRs are distributed near genes^48,57^, and tri- and hexanucleotide SSRs predominated in the coding regions of these bovids. In the study, we have demonstrated that SSRs are obvious correlated with TEs (Fig. 1E and 2E).

The birth or death of SSRs is seemingly regulated by polymerase slippage, point mutations, and other activities involving chromatin reorganization^58,59^. SSR loci have a high mutation rate (10^−6^ to 10^−2^/generation) which is due to strand slippage and unequal recombination leads to indels of repeat units^3^. The mutation rates associated with SSR loci are influenced by motif length, repeat number, and repeat type^60–63^. Mutation rates increase or decrease SSR repeat number, which are both frequent and reversible. Long SSR alleles have a downward mutation rates, which could result in a size constraint of SSRs^64–68^. Mutation rates also vary for different SSR loci within the same species^69^. There have been reported that a differential mutability rates for different SSRs occur in the genomes of two subspecies of rice^47^. Evolutionary dynamics of SSRs was regulated by their neighboring sequences^63^. SSR mutation rates vary obviously across the genomes. The abundance of tri- and hexanucleotide in coding regions also supported that specific selection against frameshift mutations in coding regions^4,22,28^. Trifold SSRs had not generated frameshifts through expansion of triplet SSRs, so that which would refrain from selective pressures in coding regions. However, non-trifold SSRs had to be subject to greater selection with the frameshift mutations^28^. RCN mutations of non-trifold SSRs in coding cause frameshifts, which can effectively inactivate gene expression or code for different or shorter protein sequences^1^. Therefore, mutation pressure contributed to the abundance of trifold SSRs in coding regions. SSR mutability per motif is relative higher at longer allele lengths^70^. Greater mutability per RCN was demonstrated in orthologous allele lengths between species^70^. These evidences have been demonstrated that SSR mutation process is great heterogeneous^70^, showing differences in mutability between different allele lengths and motif sizes and between species.

## Material and Methods

### The sequences of intragenic and intergenic regions

We selected whole genome sequences of six bovids as subjects to analyze the SSR distribution of different genomic regions. The bovid genome sequences were downloaded in FASTA format from the Ensembl (http://asia.ensembl.org/index.html) and NCBI (https://www.ncbi.nlm.nih.gov/). The sequences of the gene models, 5′UTRs, coding regions, introns, 3′UTRs, TEs, and intergenic regions were generated according to the positions in the genome annotations. The intergenic regions referred to the interval sequences between gene and gene that were not comprised of the introns, coding regions, UTRs, and other related sequences. SSRs can be grouped into six categories^23,61,71^, which were identified and scanned for SSRs of 1–6 bp using the software MSDB (Microsatellite Search and Building Database)^72^ and Krait^16^. To compare our results, the same tool and search parameters were used in the data analysis of these bovid genomes.

### SSRs identification and investigation

Since bovid species are large genomes, relatively systemic search criteria^72^ were adopted in the study. In this study, repeat units with being circular permutations and/or reverse complements of each other were grouped together as one repeat unit for statistical analysis^73,74^. For tetra- and hexanucleotide repeat units, relatively systemic combination criteria were applied^23^ in the process of filtration. For the sake of comparative analysis among different repeat types or motifs, relative abundance was determined, which means the number of SSRs per Mb of the sequence analyzed^72,75^. These total numbers have been normalized as relative abundance to allow comparison in the different genomic regions. In the four DNA bases, percentage of guanine (G) plus cytosine (C) was called GC-content in the analyzed sequence.

### Variation analysis of SSRs

In order to analyze the variation of RCN of different repeat SSR types in the different genomic regions, we introduce the CV, which the calculation formula is as follow:

$${\rm{CV}}={\rm{S}}/\bar{x}\times 100 \% .$$

where S is the standard deviation of the RCN of one SSR, $\bar{x}$ is the average of the RCN. The variation of RCN of two or more SSRs were comparative analyzed by the CV, which can eliminate the effect of different unit and mean, and is able to really reflect variation level of RCN of different SSRs.

## Electronic supplementary material

Supplementary Information
