# Supplementary material for: Distribution patterns and variation analysis of simple sequence repeats in different genomic regions of bovid genomes
Source: Sci Rep. 2018 Sep 26;8:14407. doi: 10.1038/s41598-018-32286-5 (PMC6158176; doi:10.1038/s41598-018-32286-5)
Supplement: Supplementary file 1 — Supplementary Information [file 41598_2018_32286_MOESM1_ESM.doc]

**Distribution patterns and variation analysis of simple sequence repeats in different genomic regions of bovid genomes**

**Wen-Hua Qi ^1^, Xue-Mei Jiang^2^, Chao-Chao Yan^3^,Wan-QingZhang^4^, Guo-Sheng Xiao^1^, Bi-Song Yue ^3^, Cai-Quan Zhou^5^***

^1^ College of Biology and Food Engineering, Chongqing Three Gorges University, Chongqing 404100, P. R. China

^2^College of Environmental and Chemistry Engineering, Chongqing Three Gorges University, Chongqing 404100, P. R. China

^4^College of Life Sciences, Sichuan Agricultural University, Ya′an, Sichuan Province 625014, P. R. China

^3^Key Laboratory of Bio-resources and Eco-environment (Ministry of Education), College of Life Sciences, Sichuan University, Chengdu 610064, P. R. China

^5^Key Laboratory of Southwest China Wildlife Resources Conservation (Ministry of Education), China West Normal University, Nanchong, 637009, P. R. China.

* Correspondence authors.Email: 604598775@qq.com.

Table S1. Percentage of mono- to hexanucleotide P-SSRs in the 5'UTRs of the bovid genomes

| Type | *B. taurus* | *B. mutus* | *Bu. bubalis* | *O. aries* | *C. hircus* | *P. hodgsonii* |
| --- | --- | --- | --- | --- | --- | --- |
| Size (Mb) | 1.95 | 2.82 | 7.76 | 2.81 | 3.73 | 2.43 |
| Mono- | 28.17 | 40.69 | 34.96 | 45.79 | 44.86 | 45.38 |
| Di- | 15.26 | 16.27 | 17.76 | 14.68 | 18.24 | 17.12 |
| Tri- | 44.13 | 32.91 | 35.83 | 29.35 | 29.73 | 26.92 |
| Tetra- | 8.92 | 6.87 | 7.36 | 5.68 | 4.86 | 6.35 |
| Penta- | 2.35 | 2.35 | 3.63 | 4.11 | 1.76 | 3.27 |
| Hexa- | 1.17 | 0.90 | 0.46 | 0.39 | 0.54 | 0.96 |
| Total | 100.00 | 100.00 | 100.00 | 100.00 | 100.00 | 100.00 |

Table S2. Percentage of mono- to hexanucleotide P-SSRs in the coding regions of the bovid genomes

| Type | *B. taurus* | *B. mutus* | *Bu. bubalis* | *O. aries* | *C. hircus* | *P. hodgsonii* |
| --- | --- | --- | --- | --- | --- | --- |
| Size (Mb) | 35.49 | 45.83 | 74.23 | 36.42 | 52.84 | 49.90 |
| Mono- | 4.21 | 4.42 | 1.72 | 8.11 | 5.44 | 6.10 |
| Di- | 2.19 | 1.33 | 1.78 | 6.70 | 2.15 | 1.43 |
| Tri- | 89.56 | 90.91 | 92.33 | 80.68 | 88.32 | 87.95 |
| Tetra- | 1.15 | 1.26 | 1.13 | 1.89 | 1.34 | 1.94 |
| Penta- | 0.35 | 0.19 | 0.18 | 0.24 | 0.13 | 0.36 |
| Hexa- | 2.54 | 1.89 | 2.85 | 2.38 | 2.62 | 2.22 |
| Total | 100.00 | 100.00 | 100.00 | 100.00 | 100.00 | 100.00 |

Table S3. Percentage of mono- to hexanucleotide P-SSRs in the introns of the bovid genomes

| Type | *B. taurus* | *B. mutus* | *Bu. bubalis* | *O. aries* | *C. hircus* | *P. hodgsonii* |
| --- | --- | --- | --- | --- | --- | --- |
| Size (Mb) | 822.56 | 1177.71 | 676.22 | 849.56 | 1799.66 | 1224.61 |
| Mono- | 48.87 | 47.96 | 47.36 | 47.98 | 47.22 | 45.90 |
| Di- | 22.59 | 23.14 | 23.37 | 23.95 | 23.81 | 24.54 |
| Tri- | 14.91 | 15.23 | 15.17 | 12.93 | 13.47 | 13.42 |
| Tetra- | 6.09 | 6.13 | 6.77 | 7.04 | 6.89 | 7.60 |
| Penta- | 7.38 | 7.41 | 7.15 | 7.88 | 8.35 | 8.35 |
| Hexa- | 0.16 | 0.14 | 0.18 | 0.21 | 0.26 | 0.18 |
| Total | 100.00 | 100.00 | 100.00 | 100.00 | 100.00 | 100.00 |

Supplementary Table S4. Percentage of mono- to hexanucleotide P-SSRs in the 3'UTRs of the bovid genomes

| Type | *B. taurus* | *B. mutus* | *Bu. bubalis* | *O. aries* | *C. hircus* | *P. hodgsonii* |
| --- | --- | --- | --- | --- | --- | --- |
| Size (Mb) | 7.65 | 13.21 | 20.37 | 6.83 | 18.76 | 11.94 |
| Mono- | 64.06 | 70.85 | 66.87 | 61.28 | 70.49 | 69.16 |
| Di- | 23.19 | 18.31 | 22.27 | 22.61 | 19.17 | 21.10 |
| Tri- | 5.73 | 5.00 | 4.72 | 8.34 | 4.72 | 4.70 |
| Tetra- | 4.98 | 4.46 | 4.43 | 5.75 | 3.95 | 3.68 |
| Penta- | 1.93 | 1.28 | 1.56 | 1.90 | 1.57 | 1.27 |
| Hexa- | 0.11 | 0.10 | 0.15 | 0.12 | 0.10 | 0.10 |
| Total | 100.00 | 100.00 | 100.00 | 100.00 | 100.00 | 100.00 |

Supplementary Table S5. Percentage of mono- to hexanucleotide P-SSRs in the TEs of the bovid genomes

| Type | *B. taurus* | *B. mutus* | *Bu. bubalis* | *O. aries* | *C. hircus* | *P. hodgsonii* |
| --- | --- | --- | --- | --- | --- | --- |
| Size (Mb) | 1,353.51 | 1,282.35 | 1,247.45 | 1,143.72 | 1,094.85 | 1,085.07 |
| Mono- | 70.27 | 66.90 | 62.10 | 61.28 | 59.34 | 61.51 |
| Di- | 14.18 | 15.69 | 19.79 | 19.22 | 19.83 | 18.74 |
| Tri- | 4.37 | 4.48 | 4.89 | 5.69 | 6.69 | 5.45 |
| Tetra- | 9.57 | 10.77 | 10.62 | 10.44 | 10.07 | 11.21 |
| Penta- | 1.55 | 1.96 | 2.38 | 3.01 | 3.71 | 2.80 |
| Hexa- | 0.06 | 0.19 | 0.22 | 0.36 | 0.37 | 0.29 |
| Total | 100.00 | 100.00 | 100.00 | 100.00 | 100.00 | 100.00 |

Supplementary Table S6. Percentage of mono- to hexanucleotide P-SSRs in the intergenic regions of the bovid genomes

| Type | *B. taurus* | *B. mutus* | *Bu. bubalis* | *O. aries* | *C. hircus* | *P. hodgsonii* |
| --- | --- | --- | --- | --- | --- | --- |
| Size (Mb) | 1,914.70 | 1,188.92 | 1,165.30 | 1,800.70 | 1,478.05 | 1,366.98 |
| Mono- | 44.17 | 42.68 | 41.47 | 43.20 | 42.33 | 41.76 |
| Di- | 23.60 | 24.13 | 24.00 | 24.68 | 24.43 | 24.61 |
| Tri- | 15.48 | 16.20 | 16.43 | 13.46 | 13.74 | 13.84 |
| Tetra- | 6.36 | 6.56 | 6.90 | 7.44 | 7.53 | 8.10 |
| Penta- | 10.20 | 10.28 | 10.98 | 10.94 | 11.68 | 11.47 |
| Hexa- | 0.20 | 0.16 | 0.21 | 0.27 | 0.29 | 0.22 |
| Total | 100.00 | 100.00 | 100.00 | 100.00 | 100.00 | 100.00 |

Supplementary Table S7. The AT-content (%) in the 5'UTRs, coding regions, introns, 3'UTRs,TEs, and intergenic regions of the bovid genomes

| Regions | *B. taurus* | *B. mutus* | *Bu. bubalis* | *O. aries* | *C. hircus* | *P. hodgsonii* |
| --- | --- | --- | --- | --- | --- | --- |
| 5'UTRs | 38.69 | 42.03 | 44.13 | 46.25 | 45.54 | 42.96 |
| coding regions | 46.67 | 47.27 | 46.57 | 48.91 | 47.31 | 46.40 |
| 3'UTRs | 55.13 | 56.87 | 55.97 | 57.39 | 54.82 | 57.08 |
| TEs | 57.47 | 57.17 | 57.37 | 57.28 | 57.24 | 57.24 |
| Introns | 57.63 | 58.51 | 57.09 | 59.13 | 57.87 | 58.63 |
| Intergenic regions | 58.61 | 58.22 | 58.51 | 58.58 | 58.53 | 58.16 |

Supplementary Table S8. The AT-content (%) of mono- to hexanucleotide P-SSRs in the 5'UTRs of the bovid genomes

| Type | *B. taurus* | *B. mutus* | *Bu. bubalis* | *O. aries* | *C. hircus* | *P. hodgsonii* |
| --- | --- | --- | --- | --- | --- | --- |
| Mono- | 96.20 | 98.70 | 98.04 | 93.01 | 96.70 | 96.65 |
| Di- | 64.28 | 64.35 | 61.97 | 60.19 | 63.40 | 64.63 |
| Tri- | 54.87 | 53.65 | 51.88 | 56.99 | 55.32 | 58.36 |
| Tetra- | 60.00 | 65.50 | 63.26 | 70.92 | 64.71 | 63.72 |
| Penta- | 56.17 | 62.73 | 61.84 | 60.07 | 62.26 | 62.22 |
| Hexa- | 40.74 | 41.67 | 48.89 | 55.88 | 53.85 | 56.99 |
| Total | 81.49 | 85.80 | 83.62 | 76.57 | 83.75 | 84.21 |

^a^ The numbers of nucleotides in SSRs are listed. For example: the total of the nucleotides in mononucleotide SSRs are 7647039 bp, one of which have 4215761 bp A+T and 3431278 bp C+G.

^b^ The percentage of nucleotides are shown in table.

Supplementary Table S9. The AT-content (%) of mono- to hexanucleotide P-SSRs in the coding regions of the bovid genomes

| Type | *B. taurus* | *B. mutus* | *Bu. bubalis* | *O. aries* | *C. hircus* | *P. hodgsonii* |
| --- | --- | --- | --- | --- | --- | --- |
| Mono- | 47.12 | 48.1 | 44.68 | 51.18 | 48.75 | 45.06 |
| Di- | 47.12 | 48.1 | 44.68 | 51.18 | 48.75 | 45.06 |
| Tri- | 27.36 | 35.75 | 30.53 | 33.09 | 39.36 | 35.91 |
| Tetra- | 45.37 | 38.89 | 31.17 | 47.85 | 42.28 | 34.17 |
| Penta- | 7.2 | 13.85 | 9.6 | 8 | 30 | 32 |
| Hexa- | 23.33 | 30.9 | 30.79 | 22.96 | 26.4 | 30.36 |
| Total | 29.86 | 36.06 | 31.33 | 29.42 | 38.33 | 34.43 |

Supplementary Table S10. The AT-content (%) of mono- to hexanucleotide P-SSRs in the introns of the bovid genomes

| Type | *B. taurus* | *B. mutus* | *Bu. bubalis* | *O. aries* | *C. hircus* | *P. hodgsonii* |
| --- | --- | --- | --- | --- | --- | --- |
| Mono- | 62.41 | 62.15 | 60.59 | 61.33 | 61.68 | 61.80 |
| Di- | 62.41 | 62.15 | 60.59 | 61.33 | 61.68 | 61.80 |
| Tri- | 39.69 | 40.21 | 40.17 | 41.57 | 42.26 | 41.97 |
| Tetra- | 70.25 | 71.24 | 70.22 | 71.20 | 72.84 | 73.18 |
| Penta- | 60.21 | 60.93 | 60.93 | 61.20 | 61.48 | 61.53 |
| Hexa- | 50.60 | 58.50 | 57.67 | 58.27 | 62.15 | 55.76 |
| Total | 73.67 | 73.89 | 73.12 | 71.20 | 73.06 | 72.67 |

Supplementary Table S11. The AT-content (%) of mono- to hexanucleotide P-SSRs in the 3'UTRs of the bovid genomes

| Type | *B. taurus* | *B. mutus* | *Bu. bubalis* | *O. aries* | *C. hircus* | *P. hodgsonii* |
| --- | --- | --- | --- | --- | --- | --- |
| Mono- | 96.20 | 98.70 | 98.04 | 93.01 | 96.70 | 96.65 |
| Di- | 64.28 | 64.35 | 61.97 | 60.19 | 63.40 | 64.63 |
| Tri- | 54.87 | 53.65 | 51.88 | 56.99 | 55.32 | 58.36 |
| Tetra- | 60.00 | 65.50 | 63.26 | 70.92 | 64.71 | 63.72 |
| Penta- | 56.17 | 62.73 | 61.84 | 60.07 | 62.26 | 62.22 |
| Hexa- | 40.74 | 41.67 | 48.89 | 55.88 | 53.85 | 56.99 |
| Total | 81.49 | 85.80 | 83.62 | 76.57 | 83.75 | 84.21 |

Supplementary Table S12. The AT-content (%) of mono- to hexanucleotide P-SSRs in the TEs of the bovid genomes

| Type | *B. taurus* | *B. mutus* | *Bu. bubalis* | *O. aries* | *C. hircus* | *P. hodgsonii* |
| --- | --- | --- | --- | --- | --- | --- |
| Mono- | 69.41 | 70.41 | 69.35 | 70.00 | 68.94 | 68.49 |
| Di- | 69.41 | 70.41 | 69.35 | 70.00 | 68.94 | 68.49 |
| Tri- | 64.19 | 62.19 | 61.65 | 59.07 | 55.16 | 60.30 |
| Tetra- | 77.35 | 80.35 | 78.30 | 81.29 | 81.08 | 82.63 |
| Penta- | 67.23 | 72.23 | 71.25 | 67.75 | 65.24 | 68.83 |
| Hexa- | 63.89 | 60.89 | 65.97 | 60.83 | 58.49 | 55.96 |
| Total | 89.29 | 88.29 | 86.77 | 85.52 | 84.11 | 86.03 |

Supplementary Table S13. The AT-content (%) of mono- to hexanucleotide P-SSRs in the intergenic regions of the bovid genomes

| Type | *B. taurus* | *B. mutus* | *Bu. bubalis* | *O. aries* | *C. hircus* | *P. hodgsonii* |
| --- | --- | --- | --- | --- | --- | --- |
| Mono- | 66.59 | 64.05 | 64.21 | 63.90 | 63.34 | 63.35 |
| Di- | 66.59 | 64.05 | 64.21 | 63.90 | 63.34 | 63.35 |
| Tri- | 40.08 | 40.66 | 40.66 | 42.72 | 42.93 | 42.44 |
| Tetra- | 73.11 | 73.48 | 74.56 | 74.48 | 75.14 | 75.80 |
| Penta- | 60.76 | 60.89 | 61.03 | 61.48 | 61.28 | 61.39 |
| Hexa- | 57.75 | 63.66 | 63.20 | 62.70 | 61.44 | 59.49 |
| Total | 73.79 | 72.72 | 72.75 | 71.67 | 72.14 | 72.11 |


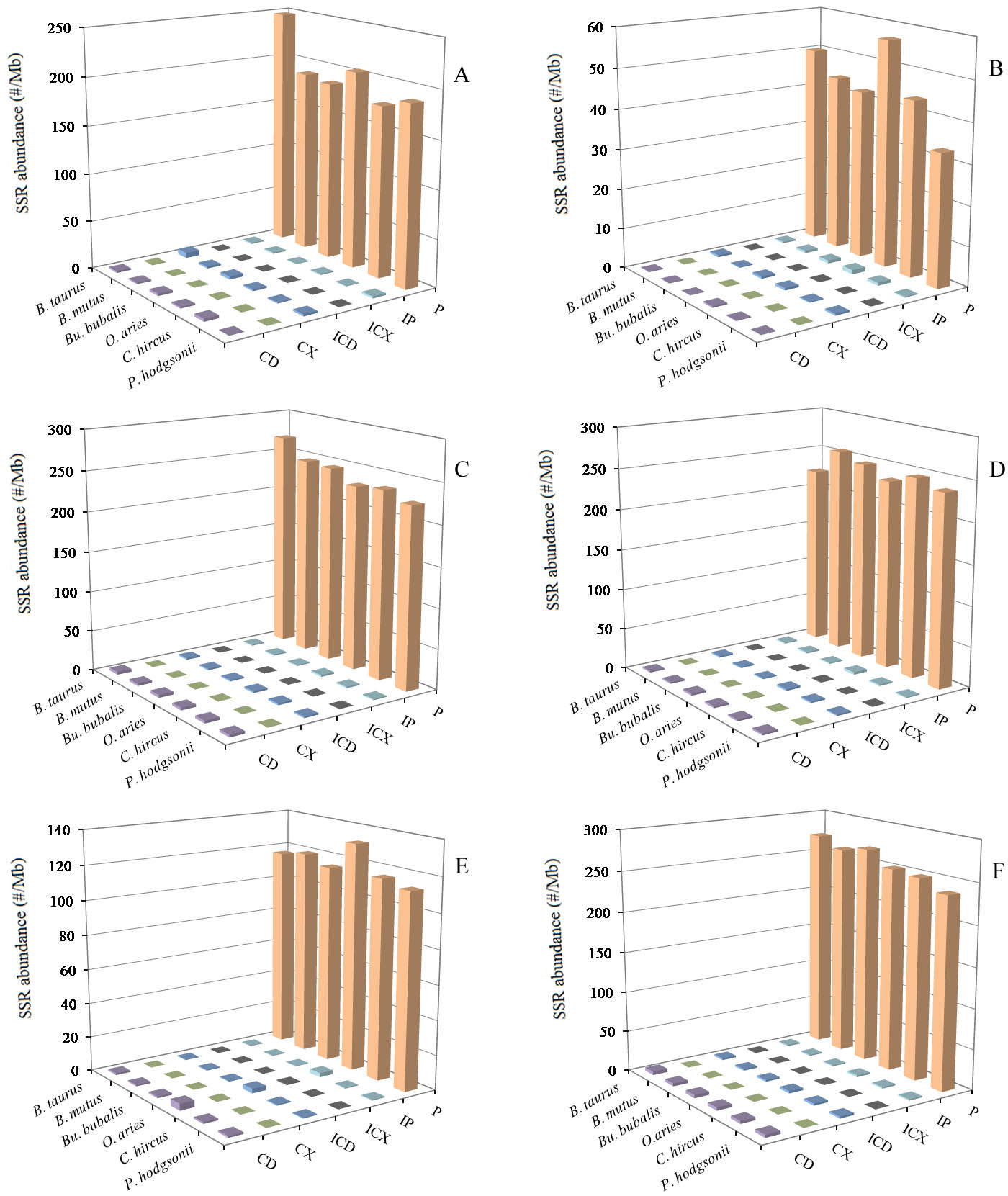


**Supplementary Fig. S1. SSRs abundance of six categories in different intragenic and intergenic regions of bovid genomes.** ABCDEF represent 5'UTRs, coding regions, introns, 3'UTRs, TEs, and intergenic regions, respectively. SSRs, Simple sequence repeats; P: Pure or perfect SSRs; IP: Interrupted perfect SSRs; CD: Compound SSRs; ICD: Interrupted compound SSRs; CX: Complex SSRs; ICX: Interrupted complex SSRs.


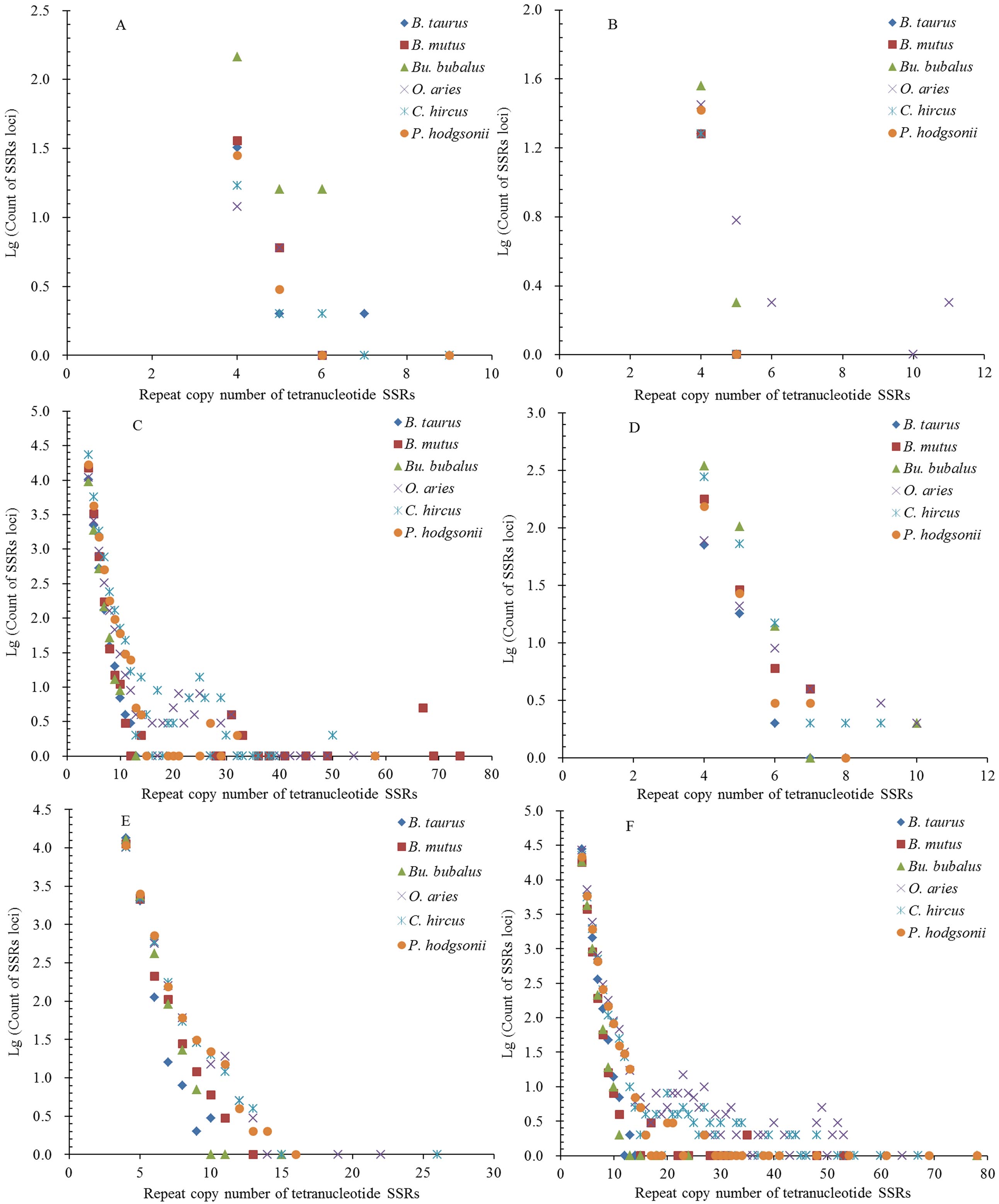


**Supplementary Fig. S2. Comparative analysis of repeat copy number (RCN) of tetranucleotide P-SSRs in different genomic regions of six bovid genomes.** ABCDEF represent 5'UTRs, coding regions, introns, 3'UTRs, TEs, and intergenic regions, respectively.


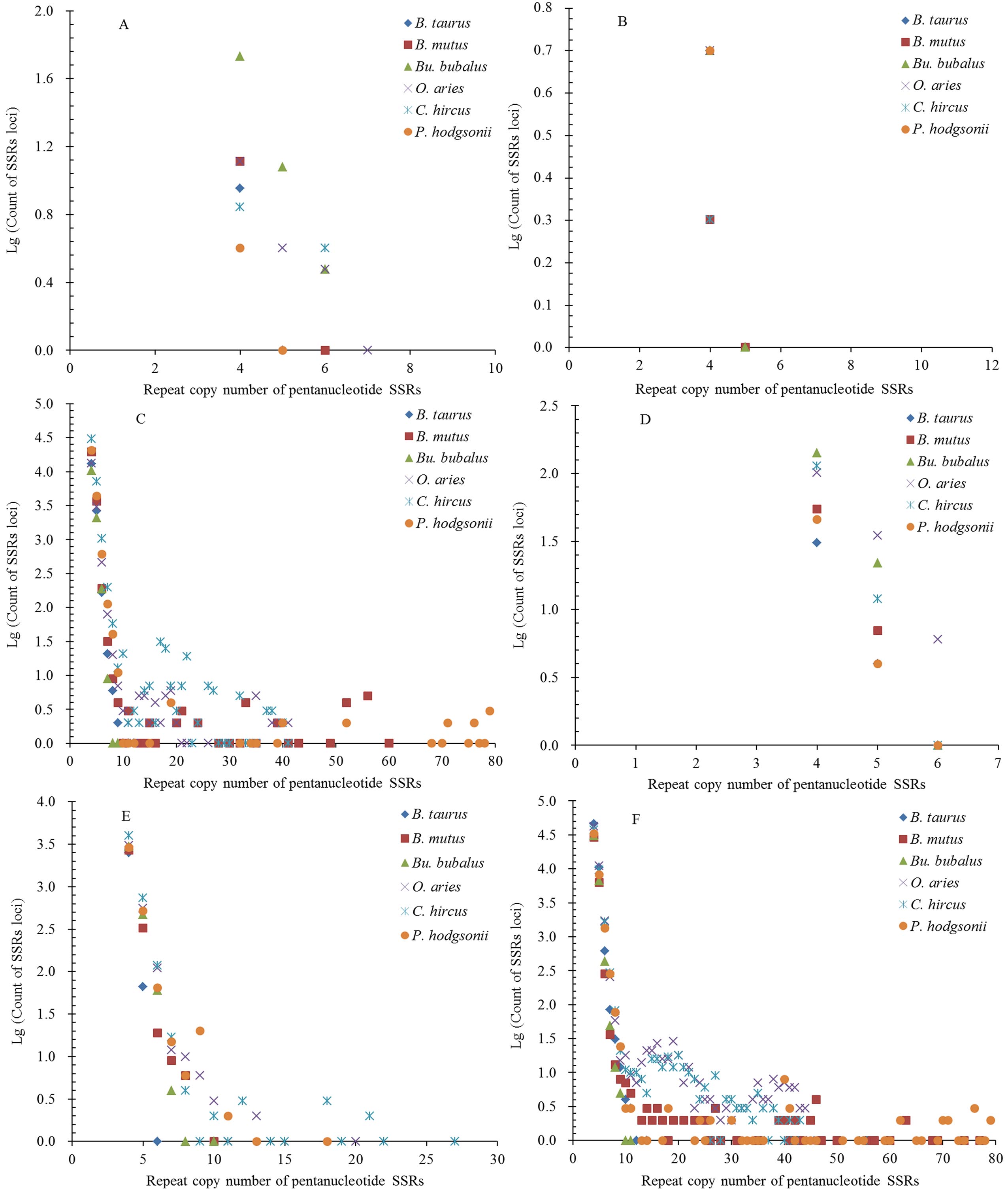


**Supplementary Fig. S3. Comparative analysis of RCN of pentanucleotide P-SSRs in different genomic regions of six bovid genomes.** ABCDEF represent 5'UTRs, coding regions, introns, 3'UTRs, TEs, and intergenic regions, respectively.

**
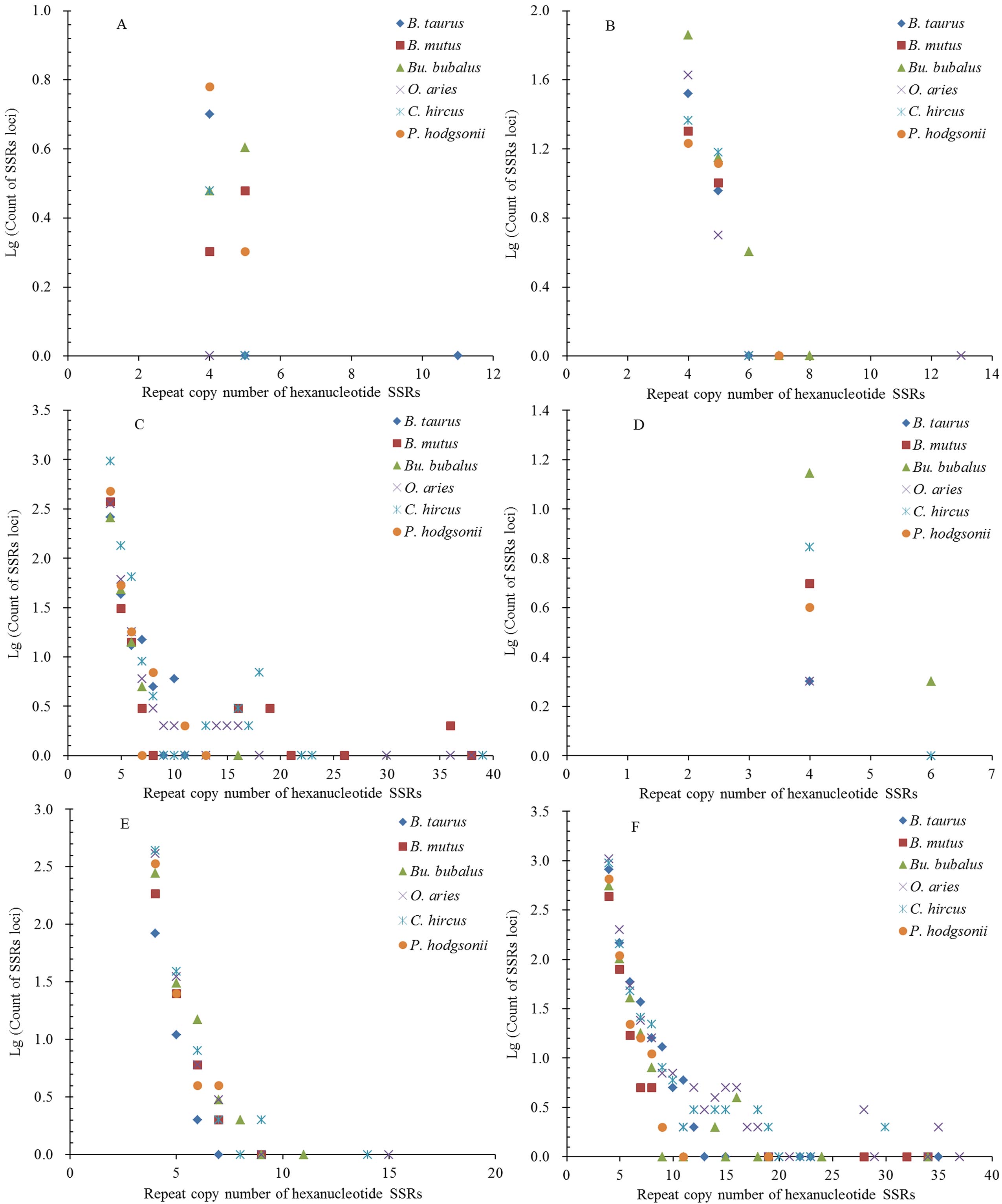
**

**Supplementary Fig. S4. Comparative analysis of RCN of hexanucleotide P-SSRs in different genomic regions of six bovid genomes.** ABCDEF represent 5'UTRs, coding regions, introns, 3'UTRs, TEs, and intergenic regions, respectively.
